# Does women’s empowerment and their socioeconomic condition affect the uptake of breast cancer screening? Findings from NFHS-5, India

**DOI:** 10.1186/s12905-022-02147-5

**Published:** 2023-01-07

**Authors:** Priti Patil, Bhakti Sarang, Prashant Bhandarkar, Rakhi Ghoshal, Nobhojit Roy, Anita Gadgil

**Affiliations:** 1 Department of Statistics, BARC Hospital, Mumbai, 400094 India; 2 Department of Surgery, Terna Medical College and Research Center, Mumbai, India; 3 Tata Institute of Social Sciences, School of Health System Studies, Mumbai, India; 4 CARE India, Okhla Industrial Estate, New Delhi, 110020 India; 5 Department of Global Public Health, Karolinska Institute, 171 77 Stockholm, Sweden; 6 The George Institute for Global Health, New Delhi, India; 7 Department of Surgery, BARC Hospital, Mumbai, 400094 India

**Keywords:** Breast cancer screening, Ecological study, Socioeconomic status, Women empowerment, National Family Health Survey

## Abstract

**Background:**

Screening for breast cancer results in early diagnosis of the disease and improves survival. However, increasing participation of women in screening programs is challenging since it is influenced by socioeconomic and cultural factors. This study explores the relationship of socioeconomic and women empowerment factors with breast cancer screening uptakes in the states and union territories of India.

**Methods:**

We used summary reports of secondary data from all the states and union territories based on the fifth wave of the National Family Health Survey in India. This ecological study compares the uptake of breast cancer screening across states of India. We considered socioeconomic status (SES) and women empowerment status (WES) indicators from the survey as independent variables and state-wise breast cancer screening uptake as dependent variables for studying their association. The determinants of breast cancer screening were calculated using a simple linear regression model.

**Results:**

We found that socioeconomic status and women empowerment status moderately correlated with breast cancer screening uptake (correlation coefficient 0.34 and 0.38, respectively). States with higher rates of literacy among women and of women who had their own bank accounts that they decided how to use reported higher uptake of breast cancer screening (p = 0.01 and 0.03, respectively). However, the correlation was not uniform across all the states. The states of Chandigarh, Delhi, Telangana, and Karnataka showed lower participation despite a higher percentage of literate women and women with their own bank accounts.

**Conclusion:**

This study indicates that women’s literacy and having their own bank account may moderately improve their participation in cancer screening. However, higher SES and WES did not translate into better screening in many of the states. More research is needed, especially for states which had low screening uptake despite relatively higher rates of women empowerment.

**Supplementary Information:**

The online version contains supplementary material available at 10.1186/s12905-022-02147-5.

## Background

In 2020 the National Cancer Registry Program (NCRP) of India estimated that one in twenty-nine Indian women would develop cancer in their lifetime. Of all forms of cancer among women in India, breast cancer is the commonest. However, only one-third of all breast cancer patients present for diagnosis at an early stage [1, 2]. To improve breast cancer survival rates, the World Health Organization (WHO) recommends cancer screening, early detection, and ensuring availability of standard referral pathways and appropriate treatment [3]. However, population-based data shows that less than 10 percent of women in India ever undergo breast examinations or participate in screening activities.

In 2010, the Ministry of Health and Family Welfare (MoHFW) in India launched the ‘National Programme for Prevention and Control of Cancer, Diabetes, Cardiovascular Diseases, and Stroke’ (NPCDCS) under the umbrella of the National Health Mission (NHM). This program includes screening for risk factors of non-communicable diseases including for three cancers viz. breast, oral, and cervical cancer. The NHM published a framework and guidance document in 2016 for the screening program to be undertaken at the primary health center level by auxiliary nurse midwives. While the Ministry of Health designs and launches programs, their implementation is the responsibility of each state. Several states have implemented the NPCDCS with financial support from the Ministry [4, 5]. In various states, the programs are also aided by regional cancer centers. In high-income countries (HICs) screening is organized by invitations to eligible population. However, in India, the guidance document focuses on strengthening screening for common risk factors for all non-communicable diseases, and advocating capacity-building of healthcare workers to perform clinical breast examination. It also advocates health promotion, education, and increasing cancer awareness among communities for accessing breast, cervix and oral cavity cancer screenings. As in 2020, 667 district-level screening cells have been set up across all 36 states of India.

To facilitate awareness and access, community health workers assess the target population for risk factors of non-communicable diseases such as hypertension, diabetes, stroke, and of common cancers such as oral, breast, and cervical during their routine household visits. However, screening for breast cancer is currently only opportunistic in all states and UTs and depends on people’s willingness to reveal the risk factors to community health workers and avail the screening services at the health centers [3, 6].

Data shows that despite these efforts put in place by the government, uptake of cancer screening among women continues to be low (< 10%) and inequitable [2]. Most common individual-level barriers to screening uptake in India, and also other LMICs, are low rates of women’s education, low income, and low awareness about screening and its benefits [7–11]. Household-level factors that affect uptake of screening include out-of-pocket expenditure from direct costs of diagnosis and treatment and indirect costs such as transport, and loss of wages during hospital visits [12]. According to the Health Belief Model or the ecological theory which shows how individual-level, household-level, community-level and systemic factors combine in complex ways to determine screening uptake, the barriers are based on psychological and behavioral factors [13]. The Fundamental Cause Theory by Link and Phelan explains that socioeconomic status is a fundamental determinant of illness and healthcare seeking. This implies that resources such as education, wealth and social connectedness are factors that enable people to adopt healthier lifestyles, make better choices for healthcare seeking and be best positioned to get least affected by illnesses [14].

Lack of autonomy among women and their low participation in household decision making, prevent women from prioritizing their health. Due to systemic gender inequities, women have to do a disproportionate share of household work, have conflicting family responsibilities, and have limited or no autonomy to spend time or money on their own health. These combine to negatively impact women’s participation in preventive healthcare or screening programs. It is thus crucial to study how women empowerment indices correlate with women’s participation in preventive health services, including screening programs in India [15–17].

Since India has wide intra-country variations in socioeconomic status, and women empowerment [18] to assess service utilization, we need to map the coverage of screening at the state level. The findings would help in the identification of areas where additional efforts to improve breast cancer screening need to concentrate on. This paper explores the relationship of socioeconomic status and women’s empowerment with uptake of breast cancer screening across the states of India. It studies the relationship using the ecological design which compares larger populations with each other rather than individual comparisons. This ecological study compares populations across the states in India using a demographic health survey called the National Family Health Survey.

## Methods

Study Setting: This ecological study is based on the fifth wave of the National Family Health Survey (NFHS-5), for which data was collected during the year 2019–2020. This periodic survey is conducted by the Ministry of Health and Family Welfare (MoHFW), Government of India, with the International Institute of Population Sciences (IIPS), Mumbai as a nodal agency for conducting the survey [19]. The NFHS is a nationally representative multi-staged survey comprising a representative sample of households from the 36 states and union territories (UTs) in India. The survey is conducted to measure various indicators related to population, health and nutrition with the objective of providing reliable and comparable datasets on health, family welfare and related issues. The state-wise summary reports of this survey provide data on indicators such as household and population characteristics, socioeconomic conditions, maternal and child health and nutrition parameters, and adult health issues, including screening for cancer. A total of 610,000 households were surveyed in the fifth wave of the NFHS.

Data Source: This study considered all the states in India as the unit of analysis (N = 36). The percentages of breast cancer screening for women aged 30 to 49 years across all 36 states and UTs in India were taken to explore the association between socioeconomic status (SES) and women empowerment status (WES) with uptake in breast cancer screening. A total of 364,556 women from this age group were interviewed.

The Government of India issued operational guidelines and advocated screening for risk factors of cancer screening in all states simultaneously in 2016 and then scaled up in a phase wise manner [6]. These survey reports from NFHS-5 do not give distinct information on screening uptake under the state or central government-run screening programs but only mention the percentages of women who have undergone screening conducted by any government, non-government or other agencies.

Study Variables: Clinical breast examination has been the recommended approach for screening and early detection for breast cancer in India by the operational guidelines from the Government of India [20]. Hence, this paper used ‘percentage of women ever undergoing breast examination’ as a broad marker for screening uptake. State and UT wise breast cancer screening percentages for women aged 30–49 years were considered as a dependent variable. SES and WES were taken to be independent variables. State specific SES and WES were derived from the household profile and women empowerment indicators as reported in the state-wise reports of NFHS-5 [21]. Additional file 1: Table S1 elaborates on the individual/component indicators used for calculating the SES and WES.

Statistical Methods: We used Dimension Indices developed by Iyengar and Sudarshan for this study [22].

Dimension Index (DI): DI is a statistical measure used to estimate the development level of a region. We calculated dimension indices for all states and union territories for each indicator by using the percentage for each indicator given in NFHS state summary reports. The value of DI lies between 0 and 1, and a greater value indicates better performance, whereas a value towards 0 indicates worse performance.

DI was calculated as follows:

$$Dimension \,Index \left( {DI} \right) = \frac{ Actual \,value \,of \,the \,indicator \,for \,a \,state - Minimum \,value \,of \,the \,indicator \,across \,all \,states/UTs}{{Maximum - Minimum \,values \,of \,the \,indicator \,across \,all \,states/UTs}}$$

For e. g. DI for Maharashtra state for breast cancer screening is calculated as:

$$DI\_Maharashtra = \frac{BCa \,Screening \,\% \,for \,Maharashtra - Minimum \,BCa \,Screening \,\% \,across \,all states \,and \,UTs}{{Maximum - Minimum \,values \,of \,the \,BCa screening \,across \,all \,states \,and \,UTs}}$$

BCa = breast cancer

Composite scores: A composite score for SES and WES was calculated by adding DI values of their respective component indicators. Table 1 shows various components of SES and WES indicators from NFHS-5 state summary reports. The DI values of breast cancer screening were categorized as low, middle and high level using the 33rd and 66th percentile marks in the range of index values. Similarly, SES and WES were categorized based on the weighted composite score according to their respective 33rd and 66th percentile values. The categories of SES and WES were cross tabulated with breast cancer screening uptake.

**Table 1:** State-wise ranking of composite scores for SES, WES and BCa screening DI for women aged between 30 and 49 years of age

State	Indicators		
	SES	WES	BC Screening
Tamilnadu	3.85	4.75	1
Puducherry	4.45	4.95	0.75
Mizoram	4.2	3.8	0.47
Kerala	4.32	3.63	0.42
Manipur	2.49	4.03	0.28
A & N Islands	3.77	3.83	0.28
Maharashtra	3.37	2.61	0.23
Goa	4.56	4.21	0.22
Andhra Pradesh	3.45	3.15	0.14
Madhya Pradesh	2.13	1.61	0.09
Meghalaya	1.68	3.28	0.09
Lakshadweep	4.04	2.85	0.09
Himachal Pradesh	3.61	3.38	0.08
Uttar Pradesh	1.96	2.05	0.07
Bihar	1.76	1.77	0.06
Punjab	3.93	3.67	0.06
Arunachal Pradesh	2.58	3.72	0.06
Tripura	2.58	1.93	0.06
Karnataka	3.44	3.73	0.06
Telangana	3.6	4.21	0.06
Haryana	3.64	2.45	0.05
Jammu & Kashmir	3.19	2.81	0.05
Delhi	4.32	3.07	0.05
Nagaland	3.1	2.93	0.05
Chhattisgarh	2.71	2.87	0.04
Odisha	1.99	3.01	0.04
DNH & DD	3.37	3.89	0.04
West Bengal	2.63	2.11	0.03
Ladakh	2.58	3.65	0.03
Rajasthan	2.49	2.17	0.03
Uttarakhand	3.41	2.83	0.03
Assam	1.65	2.39	0.03
Jharkhand	1.22	2.78	0.02
Sikkim	3.85	3.87	0.02
Gujarat	3.14	2.28	0.02
Chandigarh	4.26	3.6	0

*SES* socioeconomic status, *WES* women empowerment status, *BCa* breast cancer, *DI* dimension index, *A & N Island* Andaman & Nicobar Islands, *DNH & DD* Dadra & Nagar Haveli and Diu-Daman

First, the paper correlated composite scores of SES and WES for each state with DI values of the screening by using the Spearman rank coefficient method. It followed the standard convention of Dancey and Reidy for interpretation of Spearman coefficient values and grouped the association as either weak, moderate or strong [23]. Second, it plotted the states’ screening uptake levels by their SES and WES using a bubble graph. Third, it used a simple linear regression model to estimate the determinants of breast cancer screening uptake using the DI values of each component indicator. Effects of SES and WES variables on screening across states were examined separately.

Statistical analysis was carried out using SPSS Version 25 (SPSS Inc, Chicago, IL, USA) for Windows, R Studio and Microsoft Excel 2020. A p value below five percent was considered statistically significant.

Data quality and ethics review: Informed consent was obtained from all individual respondents before starting the survey. The privacy of data and confidentiality of respondents were maintained while conducting the survey. This paper is based on secondary data and summary indicators of the NFHS-5 survey collected by the IIPS and therefore does not require any ethics clearance. All the analysis and results are presented with an unbiased intent.

## Results

Table 1 provides details of composite scores of SES and WES and dimension index of uptake of breast cancer screening for all states and UTs. The scores are arranged in descending order of uptake. A higher score represents better uptake whereas score towards 0 represents poorer uptake. The study shows that the states of Tamil Nadu, Pondicherry, Kerala, Mizoram and Andaman & Nicobar Islands were the top five ranking states in SES and WES which had high screening uptake. (Table 1).

Additional file 2: Table S2 provides categorization of SES, WES and screening uptake as low, middle and high categories. The states of Manipur, Meghalaya and Madhya Pradesh demonstrate high uptake of breast cancer screening despite having lower SES compared to other states. The states of Assam, Jharkhand, Odisha, Rajasthan, and West Bengal have low SES and WES as well as low uptake of screening. Dadra and Nagar Haveli Diu-Daman, Sikkim and Chandigarh show low uptake of screening despite better SES and WES scores. (Additional file 2: Table S2).

This study finds a significant moderate positive correlation of composite SES (correlation coefficient: 0.336, p value = 0.045) and WES (correlation coefficient:0.378, p value = 0.023) with breast cancer screening uptake (Table 2).

**Table 2:** Association of BCa screening uptake with SES and WES in women aged 30–49 for all 36 Indian states and union territories

Indicators (composite scores)	BC screening DI	
	Spearman’s rank correlation coefficient	p value
Socioeconomic Status	0.336	0.045
Women Empowerment Status	0.378	0.023

*SES* socioeconomic status, *WES* women empowerment status, *DI* dimension index, *BCa* breast cancer

**Fig. 1:**
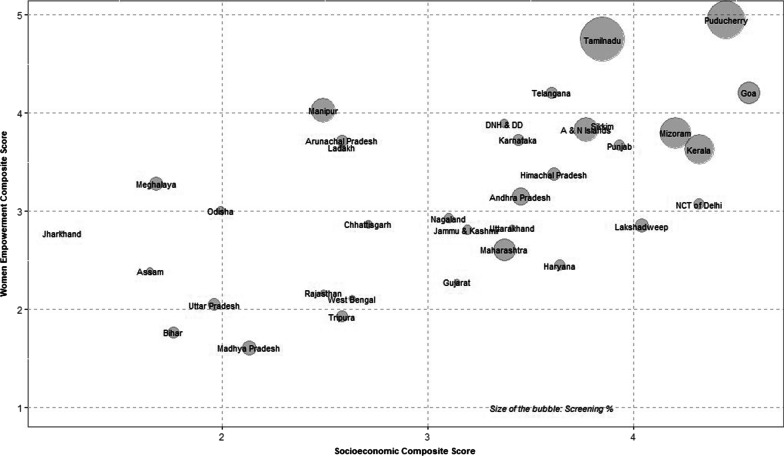
State-wise uptake of screening and socioeconomic status (SES) and women empowerment status (WES)

Figure 1 represents the state-wise screening uptake status according to SES and WES in India. The size of the bubble represents the DI values of breast cancer screening uptake in the respective states.

Table 3 shows the results of linear regression analysis. None of the SES and WES component indicators demonstrated any significant association with uptake of screening, except for literacy rate among women and percentage of women with own bank accounts that they use. For a 1-unit increase in the index for literacy rate in women, a 0.608 units increase was observed in screening uptake. Similarly, for a 1-unit increase in the index of women having a bank account that they have access to themselves, a 0.304 increase was seen in screening uptake.

**Table 3:** Determinants of uptake of breast cancer screening across 36 states and UTs using simple linear regression model

Indicators	Unstandardized Coefficient				Correlation	
	B (95% CI)	Std. Error	p value	R Square	r	p value
*Socioeconomic status indicators*						
Intercept (constant)	− 0.126 (− 0.426–0.173)	0.147	0.395	0.308		
Households with electricity	− 0.148 (− 0.478–0.183)	0.162	0.369		0.192	0.132
Households with an improved drinking-water source	0.242 (− 0.134–0.618)	0.184	0.198		0.157	0.180
Households that use an improved sanitation facility	− 0.344 (− 0.843–0.155)	0.244	0.170		0.233	0.086
Households using clean fuel for cooking	0.148 (− 0.143–0.439)	0.143	0.308		0.388	0.010
Women literacy	0.608 (0.11–1.106)	0.244	0.018		0.437	0.004
*Women empowerment status indicators*						
Intercept (constant)	− 0.335 (− 0.619–0.051)	0.139	0.023	0.473		
Women who usually participate in three household decisions	0.289 (− 0.044–0.622)	0.163	0.087		0.408	0.007
Women who were employed in the last 12 months and were paid in cash	0.238 (− 0.012–0.489)	0.122	0.062		0.406	0.007
Women owning a house/land alone or jointly with husband	− 0.063 (− 0.313–0.186)	0.122	0.608		− 0.161	0.174
Women having a bank/savings account that they themselves use	0.304 (0.017–0.591)	0.14	0.039		0.406	0.007
Women having a mobile phone that they themselves use	0.111 (− 0.153–0.375)	0.129	0.397		0.385	0.010
Women who use hygienic methods of protection during their menstrual period	0.021 (− 0.253–0.296)	0.134	0.875		0.403	0.007

*UTs* union territories

## Discussion

This ecological study analyzes association of uptake of breast cancer screening among women with socioeconomic and women empowerment indices using the NFHS-5 dataset. It finds that composite SES and WES have a mild to moderate correlation with screening, but individual indicators do not have significant correlation except for percentage of literate women and percentage of women with own bank accounts that they use. The higher SES or WES scores did not uniformly translate to higher uptake of breast cancer screening.

### Association of SES with uptake of breast cancer screening

States and UTs such as Kerala, Tamil Nadu, Puducherry, Goa, Andaman and Nicobar Islands showed high SES with high uptake of screening. The findings of this study are supported by other studies from within India and those from other continents which have found that higher SES and higher levels of women’s education correlate with higher uptake of screening [2, 11, 24–27]. A multinational study from Sub-Saharan Africa which used demographic and health survey (DHS) data, similar to the NFHS in India found that financial security from health insurance, the wealth index of the family and higher education predicted higher uptake of breast cancer screening among women [25]. This study suggested that women with lower income, women who usually earn on a per day basis, prioritized their daily wages and feeding their family over spending on preventive health services especially for themselves. This led to lower screening participation among this group. The multi-country study from Africa also documented that women with higher education levels are likely to be better informed about healthier lifestyles and cancer risk factors, and thus are more likely to present themselves for screening. The Fundamental Cause Theory proposes that improved SES enables people to access and possess resources such as money, power and education. Such individuals are thereby socioeconomically equipped to choose healthier lifestyles, which reduces risk factors alongside increasing access to treatments. This argument highlights the need to improve the outreach of education, health related awareness initiatives and financial independence among women [26]. Mishra et al. point out that prohibitive costs involved in diagnosis and treatment especially when insurance covers or universal health coverage are unavailable, become barriers to uptake of preventive healthcare such as screening [2].

### Association of WES with uptake of breast cancer screening

This study documented that women empowerment status correlated positively with uptake of screening in the states and UTs such as Kerala, Tamil Nadu, Mizoram, Puducherry Andaman and Nicobar Islands. The states and UTs with higher proportion of literate women and of women who have bank accounts that they themselves operate, had better participation in screening. Higher education and literacy are likely to make women more aware about health facilities and also convince them to prioritize their own health which in turn would lead to improved healthcare seeking behavior. Negi et al. [11] in their study of inequities in cancer screening, point out that women who were financially independent were better able to make choices regarding their own health. A qualitative study from Tamil Nadu emphasized that 50 percent of the women studied mentioned “husbands did not allow them to go for screening” [26]. This highlights that lack of women empowerment impacts their ability to make healthcare related decisions, and is very likely to lead to poor healthcare seeking behavior among women. A multicenter study from Qatar, which is a high income country, found that improved screening practices among women associated with higher income and higher education levels [27]. This suggests that improvement in women empowerment indicators and of women’s participation in decision making are necessary for increasing participation in screening activities.

### Low screening uptake despite high SES and WES

The outlier states in this study were Chandigarh, Sikkim, Delhi, Punjab and Telangana, where high SES and WES did not correlate with higher uptake of screening. This suggests that more focused efforts are needed to increase uptake of screening in addition to improving women’s literacy, empowerment and socioeconomic status. A systematic review, including Indian studies on breast cancer screening, found that women had low cancer awareness irrespective of their SES and education [24]. Focused efforts to increase breast cancer awareness even among populations where women’s literacy and SES are high, need to be designed and rolled out. Breast cancer is asymptomatic in the initial stages and there is a lack of perceived need for examination among women. Also, the reproductive risk factors for breast cancer, such as late menopause and late first pregnancy, may not necessarily be known even to women with higher education [24, 26].

The study did not deep dive into explanations for this lack of awareness but points to a need for addressing socio-cultural factors as a gap between education and cancer awareness and health seeking [24]. Embarrassment of revealing body parts to male examiners, social stigma surrounding cancer, fear of disfigurement, perceived inevitability of death once diagnosed with cancer are some of the important barriers to uptake of breast cancer screening in India [24, 26, 28, 29]. The northeastern state of Sikkim has low uptake of screening despite high SES and WES. It is likely to be due to the poor access to healthcare services women have in a state with rugged hilly terrains and very few centers offering screening and specialized cancer care [7].

## Limitations

This study has its limitations. The study does not indicate any causal relationship between WES and SES with uptake of breast cancer screening. It only supports evidence for SES and WES as contributory factors to uptake of screening among women. The association of the study variables have been estimated on the basis of the summary data available for the states and UTs. Granular individual level data on SES and WES are needed to make stronger inferences. Screening programs were implemented by the Ministry across all states at the same time but differentiated roll outs and contribution of some large-scale programs which are state specific, could have added to higher screening uptake in some states. This study was unable to quantify those effects. This may also explain the fact that association of SES and WES was not uniform throughout the states. Some states like Manipur and Meghalaya had higher uptake of screening despite low SES and WES. In addition, women’s religion and marital status are other known determinants that this study did not consider due to lack of individual level data at the time of conducting this study [17]. This study also has limitations inherent to survey-based studies in which self-reporting errors cannot be eliminated.

## Conclusion

This ecological study suggests that improvements in socioeconomic status (SES) and women empowerment status (WES) enhance breast cancer screening uptake in India. However, high SES and WES does not naturally translate into better screening in many states. However, individual indicators such as women's literacy and their financial independence have significant correlation with uptake of breast cancer screening. Other individual-level factors such as awareness about breast cancer symptoms, cultural factors, and cancer stigma also need to be considered before a stronger correlation can be made between uptake of screening and SES and/or WES. The implementation of organized breast cancer awareness and screening programs may improve the screening attendance in future.

## Supplementary Information

**Additional file 1. Table S1.**. Indicators used to calculate the socioeconomic status (SES) and women empowerment status (WES).

**Additional file 2. Table S2.** Stratified socioeconomic composite scores and women empowerment composite scores with breast cancer screening dimension index.

## Data Availability

The analysis is based on the published summary of the fifth wave of National Family Health Survey. It is available on http://rchiips.org/nfhs/factsheet_NFHS-5.shtml.

## Abbreviations

BCa: Breast cancer
SES: Socioeconomic status
WES: Women empowerment status
DI: Dimension index
NFHS: National Health Family Survey
NPCDCS: National Programme for Prevention and Control of Cancer, Diabetes, Cardiovascular Diseases and Stroke
