# Supplementary material for: Does women’s empowerment and their socioeconomic condition affect the uptake of breast cancer screening? Findings from NFHS-5, India
Source: BMC Womens Health. 2023 Jan 7;23:7. doi: 10.1186/s12905-022-02147-5 (PMC9824936; doi:10.1186/s12905-022-02147-5)
Supplement: Supplementary file 1 — Additional file 1. Table S1.. Indicators used to calculate the socioeconomic status (SES) and women empowerment status (WES). [file 12905_2022_2147_MOESM1_ESM.docx]

**Supplementary Table 1**: I**ndicators used to calculate the socioeconomic status (SES) and women empowerment status (WES)**

| **Components of Socioeconomic Status** | **Components of Women Empowerment Status** |
| --- | --- |
| Population living in the households with electricity | Currently married women usually participate in three household decisions (Decision about health care of herself, making major household purchase, and visits to her family and relatives.) |
| Population living in households with an improved drinking-water source | Women who were employed worked in the last 12 months and were paid in cash |
| Population living in households that use an improved sanitation facility | Women owning a house/land alone or jointly with husband |
| Households using clean fuel for cooking | Women having a bank/savings account that they themselves use. |
| Women who are literate | Women having a mobile phone that they themselves use independently. |
|  | Women who use hygienic methods of protection during their menstrual period |

Source: Guide to DHS statistics (21)
