# Supplementary material for: Does women’s empowerment and their socioeconomic condition affect the uptake of breast cancer screening? Findings from NFHS-5, India
Source: BMC Womens Health. 2023 Jan 7;23:7. doi: 10.1186/s12905-022-02147-5 (PMC9824936; doi:10.1186/s12905-022-02147-5)
Supplement: Supplementary file 2 — Additional file 2. Table S2. Stratified socioeconomic composite scores and women empowerment composite scores with breast cancer screening dimension index. [file 12905_2022_2147_MOESM2_ESM.docx]

**Supplementary Table 2: Stratified socioeconomic composite scores and women empowerment composite scores with breast cancer screening dimension index**

| **Group** | **Breast Cancer Screening DI** | | | |
| --- | --- | --- | --- | --- |
|  | **Low** | **Middle** | **High** |  |
| Socioeconomic composite score | | | | |
| Low | Assam, Jharkhand, Odisha, Rajasthan, West Bengal | Arunachal Pradesh, Bihar, Tripura, Uttar Pradesh | Madhya Pradesh, Manipur, Meghalaya |  |
| Middle | Chhattisgarh, Dadra & Nagar Haveli and Daman-Diu, Gujarat, Ladakh, Uttarakhand | Haryana, Himachal Pradesh, Jammu & Kashmir, Karnataka, Nagaland | Andhra Pradesh, Maharashtra |  |
| High | Chandigarh, Sikkim | Delhi, Punjab, Telangana | Andaman & Nicobar Islands, Goa, Kerala, Lakshadweep, Mizoram, Puducherry, Tamil Nadu |  |
| Women empowerment composite score | | | | |
| Low | Assam, Chhattisgarh, Gujarat, Jharkhand, Rajasthan, West Bengal | Bihar, Haryana, Tripura, Uttar Pradesh | Madhya Pradesh, Maharashtra |  |
| Middle | Chandigarh, Odisha, Uttarakhand | Himachal Pradesh, Jammu & Kashmir, Nagaland, Delhi | Andhra Pradesh, Kerala, Lakshadweep, Meghalaya, Mizoram |  |
| High | Dadra & Nagar Haveli and Diu-Daman, Ladakh, Sikkim | Arunachal Pradesh, Karnataka, Punjab, Telangana | Andaman & Nicobar Islands, Goa, Manipur, Puducherry, Tamil Nadu |  |

DI: Dimension Index
